# Lesion correlates of auditory sentence comprehension deficits in post-stroke aphasia

**DOI:** 10.1016/j.ynirp.2021.100076

**Published:** 2022-01-04

**Authors:** Erica Adezati, Melissa Thye, Amelia J. Edmondson-Stait, Jerzy P. Szaflarski, Daniel Mirman

**Affiliations:** a Department of Psychology, University of Edinburgh, Edinburgh, UK; b Centre for Clinical Brain Sciences, University of Edinburgh, Edinburgh, UK; c Department of Neurology and the University of Alabama at Birmingham Epilepsy Center, University of Alabama at Birmingham Heersink School of Medicine, Birmingham, AL, USA

**Keywords:** Aphasia, Language comprehension, Lesion-symptom mapping

## Abstract

Auditory sentence comprehension requires coordination of multiple levels of processing: auditory-phonological perception, lexical-semantic comprehension, syntactic parsing and discourse construction, as well as executive functions such as verbal working memory (WM) and cognitive control. This study examined the lesion correlates of sentence comprehension deficits in post-stroke aphasia, building on prior work on this topic by using a different and clinically-relevant measure of sentence comprehension (the Token Test) and multivariate (SCCAN) and connectome-based lesion-symptom mapping methods. The key findings were that lesions in the posterior superior temporal lobe and inferior frontal gyrus (pars triangularis) were associated with sentence comprehension deficits, which was observed in both mass univariate and multivariate lesion-symptom mapping. Graph theoretic measures of connectome disruption were not statistically significantly associated with sentence comprehension deficits after accounting for overall lesion size.

## 1 Introduction

Auditory sentence comprehension requires the coordination of multiple levels of processing: auditory-phonological perception, lexical-semantic comprehension, syntax parsing and discourse construction, as well as executive functions such as verbal working memory (WM) and cognitive control. The phonological and lexical aspects appear to be supported by a “ventral stream” of regions in the lateral temporal lobe (e.g., Hickok and Poeppel, 2007), but the critical regions are less clear at the sentence level. Left inferior frontal cortex, inferior parietal cortex, and posterior superior temporal cortex are the most consistently implicated regions for sentence-level comprehension deficits (for a review see Wilson, 2017).

Early voxel-based lesion-symptom mapping (VLSM) studies used composite comprehension scores and found that posterior middle temporal gyrus (pMTG) damage was most strongly associated with comprehension deficits (Bates et al., 2003; Dronkers et al., 2004), possibly extending dorsally into angular gyrus (AG) and anteriorly to the anterior temporal lobe (ATL) and inferior frontal gyrus (IFG). Subsequent VLSM studies have confirmed that pMTG and AG damage are associated with sentence comprehension deficits (Kristinsson et al., 2020; Pillay et al., 2017). Some of these studies have focused on so-called agrammatic sentence comprehension, which is characterised by substantially worse performance on unusual (“noncanonical”) sentence structures compared to more typical and familiar (“canonical”) sentence structures and is traditionally associated with Broca’s area damage (e.g., Grodzinsky and Santi, 2008). However, both canonical and noncanonical sentence comprehension appear to be associated with temporo-parietal damage (Rogalsky et al., 2018; Thothathiri et al., 2012). Lwi et al. (2021) compared three auditory comprehension tasks and found that pMTG damage was associated with deficits in all three tasks, along with differences between tasks: the lesion correlates of single word comprehension extended into inferior temporal regions and the lesion correlates of sequential commands extended into superior temporal and angular gyri.

Rogalsky et al. (2018) noted that damage to inferior frontal cortex was significantly associated with response bias in sentence plausibility judgments, suggesting that IFG may be involved in cognitive demands related to sentence comprehension. One important factor is working memory (WM; which these studies seem to use synonymously with short-term memory [STM], although the two terms are not exactly equivalent in cognitive psychology). WM/STM is important for sentence comprehension because sentence input is a sequence of words that must be temporarily maintained in memory while constructing a holistic – usually hierarchical – representation of the meaning. Several studies have found that WM/STM deficits are associated with sentence comprehension deficits, and that both are associated with parietal-frontal damage (Barbey et al., 2014; Leff et al., 2009; Newhart et al., 2012).

The present study provides a useful converging perspective on sentence comprehension deficits. First, sentence comprehension was measured using the Token Test (De Renzi and Vignolo, 1962), which, to our knowledge, has not been used in prior lesion-symptom mapping studies. The sentences on the Token Test do not use non-canonical structures or complex embeddings, which allows an investigation of sentence comprehension decoupled from complex syntactic processing. The Token Test sentences also use a relatively restricted set of highly familiar concepts (colours, shapes, spatial and temporal relations), so deficits are unlikely to arise from impaired comprehension of individual words (i.e., lexical-semantic deficits).

However, the Token Test does require combinatorial processing in the form of maintaining and executing a sequence of movements. These maintenance and sequencing operations should rely on verbal WM. They may also require semantic control because repetition of items from semantic categories tends to produce competition that requires control resources to resolve, as seen in blocked cyclic naming and in “access” deficits more generally (Mirman and Britt, 2014). The Token Test is also used in clinical contexts to assess auditory sentence comprehension, so this study may provide helpful information for interpreting its results in clinical settings.

Second, there is growing concern about reproducibility, particularly regarding brain-behaviour relationships (e.g., Boekel et al., 2015), but direct replication of lesion-symptom studies is generally both practically and financially impossible (for more discussion see Geller et al., 2019). By using a different measure of sentence comprehension the present study provides a conceptual replication of prior LSM studies of sentence comprehension (particularly of Lwi et al., 2021, and Pillay et al., 2017). It further does this using fully reproducible methods and a publicly available data set (https://osf.io/br3dm).

Third, since sentence comprehension requires coordinating multiple processes, it may well rely on a network of non-contiguous regions. Standard mass-univariate VLSM methods are not very well-suited to detecting such networks. Thus, we supplement those analyses with a more recently developed multivariate LSM method based on sparse canonical correlations (SCCAN; Pustina et al., 2018), which is better able to detect when damage to multiple regions contribute to a deficit (for recent applications and additional discussion of the advantages of SCCAN see Thye et al., 2021; Thye and Mirman, 2018). To characterise the lesion impact on information transfer within networks, we further include graph-theoretical analyses of whether sentence comprehension deficits are associated with white matter connectivity disruption.

## 2 Methods

### 2.1 Data

The data were drawn from two large-scale studies of language processing following left hemisphere stroke. Detailed descriptions of the study design, participants, and neuroimaging protocols have been provided in previous studies (Allendorfer et al., 2012, 2021; Griffis et al., 2017; Nenert et al., 2017; Szaflarski et al., 2021). MRI data were collected using Philips 3T or Siemens 3T scanners. On Philips 3T scanner, the high-resolution T1-weighted anatomical scans were acquired using a magnetization prepared - rapid gradient echo (MPRAGE) acquisition with the following parameters: TR/TE = 8100/3.7 ms, FOV = 25.0 × 21.0 × 18.0 cm, matrix = 252 × 211, flip angle = 8°, slice thickness = 1 mm. On Siemens Allegra 3T scanner, the corresponding scan had the following parameters: TR/TE = 2300/2.17 ms, FOV = 25.6 × 25.6 × 19.2 cm, matrix = 256 × 256, flip angle = 9°, slice thickness = 1 mm. Finally, the anatomical scan from Siemens Prisma 3T scanner used the following sequence: TR/TE = 2300/3.37 ms, FOV = 25.6 × 25.6 × 19.2 cm, matrix = 256 × 256, flip angle = 9°, slice thickness = 1 mm. The diffusion weighted imaging sequence parameters are provided in Table 2. Almost all of the participants included in the current study were previously included in a lesion-symptom mapping study of semantic and phonological fluency (Thye et al., 2021).^1^

**Table 1:** Participant Demographic Information.

	Mean (SD)	Range
Age (years)	52.14 (15.12)	22.65–84.83
Lesion Size (cc)	90.97 (64.65)	1.88–262.83
Time Since Stroke (months)	38.87 (38.09)	2.24–167.93
Token Test	24.36 (13.22)	4–44
PPVT	189.35 (28.23)	75–223
BNT	33.88 (20.25)	0–60
SFT	19.12 (15.90)	0–62
COWAT	8.44 (8.64)	0–36
Gender (M:F)	33:17	
Handedness (R:L:A)	44:4:2	

*Note.* SD, standard deviation of the mean; cc, cubic centimetres; PPVT, Peabody Picture Vocabulary Test; BNT, Boston Naming Test; SFT, Semantic Fluency Test; COWAT, Controlled Oral Word Association Test; M, Male; F, Female; R, Right; L, Left; A, Ambidextrous.

**Table 2:** Diffusion weighted imaging parameters.

Sequence	1	2	3
N	25	18	7
Scanner (3T)	Philips Achieva	Siemens Allegra	Siemens Prisma
Voxel Size (mm)	1.9 × 1.9 × 2.38	2.5x2.5x2.5	2x2x2.6
FOV (mm)	180x161	240x240	244x244
TR (ms)	9403	9400	5500
TE (ms)	69	89	69
Flip angle	90	90	90
Sampling directions	32	32	64
*b*-Value (s/mm^2^)	800	800	1000

*Note.* N, number of participants; 3T, 3 T; mm, millimetre; ms, millisecond.

Prospectively collected Magnetic Resonance Imaging (MRI) and psycholinguistic data from 50 participants with aphasia secondary to a single left hemisphere stroke were analysed. Analysis of these de-identified data was approved by the Institutional Review Board at the University of Alabama at Birmingham (IRB-120726004, IRB-120726006) and the PPLS Research Ethics panel at the University of Edinburgh (Ref No. 16–2021/2). Participant demographic information is presented in Table 1.

### 2.2 Auditory sentence comprehension measure

The behavioural measure for the present analyses was the Token Test (De Renzi and Vignolo, 1962), which requires participants to manually carry out the examiner’s verbal commands involving a set of tokens differing in shape, size and colour. The verbal instructions include simple commands (e.g., ‘Pick up the yellow rectangle’) as well as more complex ones (e.g., ‘Before touching the yellow circle, pick up the red rectangle’). Scores between 37 and 40 are thought to indicate a mild language impairment, scores ranging between 17 and 36 a moderate deficit, and scores less than 17 indicate severe comprehension deficits (as in previous work: Szaflarski et al., 2015). As shown in Table 1, the participants in this sample had a very broad range of time since stroke (2 months - 14 years); however, time since stroke was uncorrelated with Token Test performance (r = -0.065, p > 0.65), so it was not included in the statistical analyses.

### 2.3 Neuroimaging measures

**Fig. 1:**
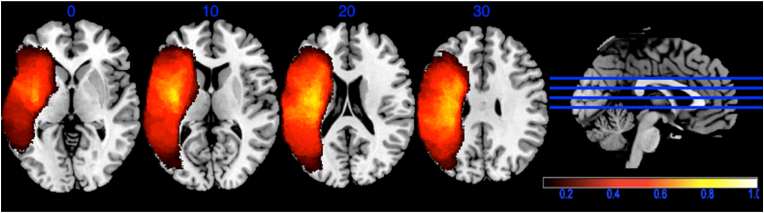
Lesion overlap map. Only voxels that were lesioned in at least 10% of the participants were included in the analyses. Colours indicate the proportion of participants with lesion in that voxel.

Automated lesion segmentation using the LINDA package in R (Pustina et al., 2016) was used to distinguish lesioned and spared brain tissue. The lesion masks were binarized and spatially normalized to the same stereotaxic space (Colin27) using symmetric normalization (Avants et al., 2008) as implemented within LINDA. After segmentation, the resulting lesion files were visually examined, and reproducible modifications were made to all lesion masks to account for consistent errors in segmentation (e.g., identifying distal clusters of healthy tissue or portions of the cerebellum as part of the lesion territory). Fig. 1 shows the lesion overlap map.

Whole-brain structural connectomes were generated from each participant’s diffusion images using probabilistic tractography. To enable fibre tracking between anatomic atlas regions, participant T1-weighted images were normalized to MNI space using the Clinical Toolbox (Rorden et al., 2012), which accounts for deformations caused by brain lesions, and the inverse transformation matrix was used to transform the AAL3 atlas (Rolls et al., 2020) into native space. The probabilistic grey and white matter tissue maps, lesion mask, and AAL atlas regions were then linearly co-registered to diffusion space using the B0 image as a reference. Where necessary, minor modifications were made to the diffusion pre-processing pipeline (i.e., selection of b0 images) to account for slight differences in scan sequences across participants.

Diffusion data were distortion corrected for motion and eddy currents, and diffusion parameters within each voxel were estimated using FSL FDT’s bedpost (Behrens et al., 2007; Jenkinson et al., 2012). Probabilistic fibre tracking was performed in diffusion space using FDT’s probtrackX with default tracking parameters (i.e., 5000 individual streamlines drawn in each voxel, 2000 maximum number of steps, 0.5 mm step length, 0.2 curvature threshold, 0.01 fiber volume threshold) and distance correction to quantify the number of streamlines connecting the atlas regions correcting for the distance between the regions. Tractography was restricted to the probabilistic white-matter map excluding the stroke lesion, and streamlines were seeded from each cortical region. The resulting matrices were corrected for region volume by dividing the number of streamlines between two regions by the combined volume of the connected regions. For each participant, this resulted in a 166x166 symmetric matrix describing the number of pairwise streamlines corrected for the distance between and size of the connected regions.

Connectivity disruption was quantified with graph theoretical measures calculated in MATLAB 2020b (*MATLAB*, 2020) using the Brain Connectivity Toolbox (Rubinov and Sporns, 2010). For measures of community structure (i.e., transitivity and clustering coefficient), input connectomes were normalized and global network metrics were calculated by taking the mean of the weighted node-level measures. For distance-based measures (i.e., characteristic path length and global efficiency), the normalized connectomes were converted to weighted distance matrices prior to calculation. The measures were generated for a whole-brain network and a left hemisphere language network defined based on prior work using similar methods (Del Gaizo et al., 2017; den Ouden et al., 2019; Fridriksson et al., 2018; Gleichgerrcht et al., 2017; Hula et al., 2020; Marebwa et al., 2017) and consisting of the following regions: middle frontal gyrus, inferior frontal gyrus (pars opercularis, pars triangularis, and pars orbitalis), precentral gyrus, postcentral gyrus, insula, temporal pole (superior and middle portions), inferior temporal gyrus, middle temporal gyrus, superior temporal gyrus, supramarginal gyrus, and angular gyrus.

Four graph theoretical measures were selected for analyses:

(1) *Average clustering coefficient:* the average of the clustering coefficients of every node within a network. Clustering coefficient is a measure of network segregation, capturing the extent to which functionally related regions are densely connected into specialized clusters. A high clustering coefficient is indicative of a network of densely connected clusters that is robust to damage (i.e., enough connections remain for information to travel to functionally related regions). Damage within the densely connected clusters can impact the efficiency of local processing (Johnson et al., 2020; Kiran et al., 2019).
(2) *Transitivity*: an alternative to average clustering coefficient for measuring network segregation. Rather than calculating node-level clustering coefficients and averaging them across the network, transitivity is defined at the network level so it is less influenced by individual nodes that have very few connections (Newman, 2003; Rubinov and Sporns, 2010). Transitivity has been found to be associated with better verbal fluency performance in normal aging (Gonzalez-Burgos et al., 2021).
(3) *Characteristic path length*: the average of the shortest paths between all pairs of nodes within the network. Characteristic path length is a measure of network integration based on how many steps are required to get from one node to another node. Higher values indicate that (on average) information must travel through more steps to get from one node to another, suggesting a less functionally integrated network (Rubinov and Sporns, 2010). It is associated with reduced functional connectivity (Griffis et al., 2020) and the closely related measure of “propagation speed” has been found to be associated with WAB aphasia quotient and fluency (Del Gaizo et al., 2017).
(4) *Global Efficiency*: the average of each individual node’s global efficiency, which is the average of inverse shortest path lengths between that node and all other nodes. Global efficiency is a measure of network integration that captures how quickly information can travel between brain regions (Latora and Marchiori, 2001). Global efficiency is conceptually closely related to characteristic path length, but the difference in their calculation has substantial implications. If two nodes are not connected, the path length between them is infinite and the communication efficiency is 0. When calculating the average, these values have radically different impact. More generally, characteristic path length is strongly influenced by long paths (infinite path length between disconnected nodes is an extreme case) but efficiency is more sensitive to short paths. This difference may be further exaggerated when these measures are calculated for stroke-damaged brains, which are more likely to have disconnected nodes or to rely on longer paths to communicate around the lesion territory. Johnson et al. (2020) found that higher global efficiency within the semantic network was associated with better response to naming therapy in chronic post-stroke aphasia.

### 2.4 Statistical analysis

Lesion-symptom mapping analyses were conducted in R (version 3.5.1) (R Development Core Team., 2018) using the LESYMAP package (version 0.0.0.9220) (Pustina, 2019). The R code used for all analyses is provided at https://osf.io/br3dm. The analyses were corrected for sufficient lesion involvement by only including voxels that were lesioned in at least 10% of the participants (minimum n = 5). All analyses were also corrected for lesion size using direct total lesion volume control (dTLVC) (Mirman et al., 2015). Multivariate LSM was conducted using SCCAN with 4-fold cross-validation to optimise sparseness (Pustina et al., 2018).

Mass-univariate LSM results were corrected for multiple comparisons using continuous permutation-based Family Wise Error Rate (FWER) at p < 0.05, with v = 100, meaning that there was a less than 5% chance of observing more than 100 false positive voxels (Mirman et al., 2018). Continuous permutation-based FWER is an extension of traditional permutation-based FWER correction that aligns with the interpretation of VLSM results, which is typically based on groups of voxels (clusters) rather than on single voxels (Mirman et al., 2018). LSM results were labelled using the Automated Anatomical Atlas (AAL) (Tzourio-Mazoyer et al., 2002) and the Johns Hopkins University (JHU) white matter atlas (Hua et al., 2008; Mori et al., 2005).

Connectivity disruption effects were tested using multiple regression to assess different measures of connectivity disruption. Several of the connectivity measures had skewed or bimodal distributions, so the analyses used robust standard error estimation (Savalei, 2014) implemented in the lavaan package version 0.6–8 (Rosseel, 2012). Separate regressions were run for metrics based on the whole brain network and the language sub-network, with overall lesion volume and the five graph theory connectivity metrics entered simultaneously in each regression.

## 3 Results

**Fig. 2:**
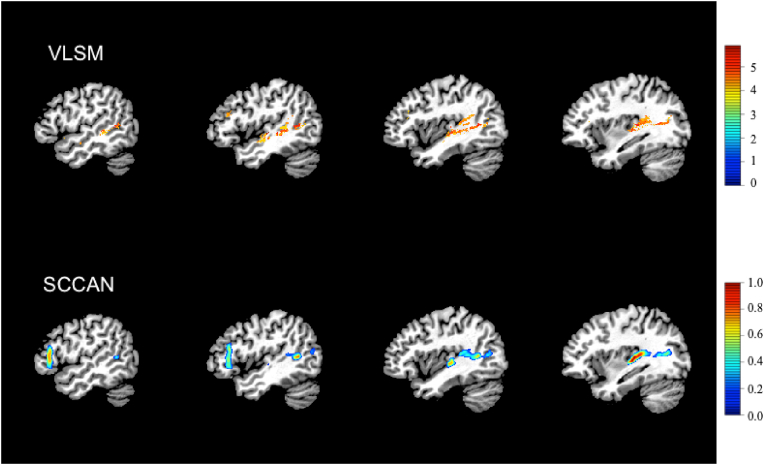
Lesion-symptom mapping (LSM) results. Top row shows suprathreshold voxels from mass-univariate VLSM results. Bottom row shows SCCAN LSM solution.

LSM results are shown in Fig. 2. The mass-univariate VLSM identified 4142 suprathreshold voxels, primarily in the posterior superior temporal lobe (STG, MTG, and Heschl’s gyrus) and a smaller cluster in IFG pars triangularis. Multivariate SCCAN LSM also identified a relatively sparse solution (optimal sparseness = 0.173, CV correlation = 0.55, *p* < 0.0001) with suprathreshold voxels in posterior superior temporal cortex, IFG pars triangularis, and frontal white matter (superior and anterior corona radiata).

The connectivity disruption regressions revealed only one statistically significant effect: overall lesion volume (*p* < 0.001 for both analyses). None of the graph theory metrics of connectivity disruption were statistically significant predictors of Token Test performance, regardless of whether they were defined at the whole brain level or for the language sub-network (all *p* > 0.2). See Table 3 for full parameter estimates, standard errors, and confidence intervals.

**Table 3:** Results of connectivity disruption regression analyses. Values are the coefficient estimates with standard errors in parentheses and 95% confidence intervals in brackets. Only lesion volume was statistically significantly associated with Token Test performance.

Metric	Whole Brain Network	Language Network
Lesion volume (cc)	−0.111 (0.024), [-0.158, -0.064]	−0.116 (0.03), [-0.174, -0.057]
Average Clustering Coefficient	6811 (6773), [-6463, 20085]	−20.7 (156), [-327, 286]
Transitivity	−4616 (6433), [-17224, 7992]	133 (136), [-134, 400]
Characteristic path length	0.011 (0.021), [-0.031, 0.053]	0.001 (0.003), [-0.004, 0.007]
Global efficiency	−202.4 (187.2), [-569.3, 164.5]	−14.9 (34.8), [-83.1, 53.2]

## 4 Discussion

This study examined the lesion correlates of sentence comprehension deficits in post-stroke aphasia building on prior work on this topic by using a different and clinically-relevant measure of sentence comprehension (the Token Test) and multivariate (SCCAN) and connectome-based lesion-symptom mapping methods. The key findings were that lesions in the posterior superior temporal lobe and IFG pars triangularis were associated with sentence comprehension deficits, which was observed in both mass univariate and multivariate LSM. These regions are often damaged separately (there was only a weak correlation between damage to frontal and temporal clusters: r ≈ 0.3) and, because stroke lesions tend to be contiguous, damage to both the anterior and posterior clusters would tend to also involve damage to the regions between them. That these intermediate regions were *not* identified by the LSM analysis suggests that the frontal and temporal regions make independent contributions to sentence comprehension deficits. Graph theoretic measures of connectome disruption were not statistically significantly associated with sentence comprehension deficits after accounting for overall lesion size.

These results converge with prior work that identified posterior superior temporal and inferior frontal regions as critical for sentence comprehension (for a review see Wilson, 2017). The Token Test is particularly similar to the sequential commands task and the present LSM results closely replicate the results reported by Lwi et al. (2021), though they did not also find a frontal effect. Lwi et al. also used both mass-univariate and multivariate LSM (finding very good convergence between them), though they used a different multivariate algorithm: SVR-LSM. Unlike SCCAN LSM, SVR-LSM requires correction for multiple comparisons (Sperber et al., 2019), which can produce artifactually focal results (Thye and Mirman, 2018), so that is one possible reason for this difference. It is also possible that the frontal contribution to sentence comprehension is inconsistent across participants. Note that Lwi et al. had a much larger sample size (N = 168) than the present study (N = 50).

We did not observe involvement of anterior temporal regions, perhaps because the semantic demands were quite limited. Nor did we observe involvement of inferior parietal regions, perhaps because the demands for hierarchical syntactic processing were limited (Matchin and Hickok, 2020). Rather, the critical regions were ones associated with speech perception, verbal WM, and semantic control, suggesting that these are the primary drivers of Token Test performance. The Token Test is also used in clinical contexts, so in addition to providing further insight into the neural correlates of sentence comprehension deficits, these results are relevant for interpreting Token Test results in clinical settings.

In sum, the present study examined auditory sentence comprehension deficits in post-stroke aphasia using new methods: a clinically-relevant behavioural measure, multivariate LSM, and connectome disruption measures. The results largely converge with prior work, highlighting the critical role of posterior superior temporal and inferior frontal regions in sentence comprehension, and providing a valuable (and rare) replication of those effects. The results also suggest more specialized roles for anterior temporal and inferior parietal regions as well as caution regarding use of connectome disruption measures.

## Declaration of competing interest

The authors declare that they have no known competing financial interests or personal relationships that could have appeared to influence the work reported in this paper.

## Footnotes

1 Thye et al. (2021) did not use any measures derived from diffusion imaging, so 6 participants with only T1 lesion maps and no diffusion data were included in the earlier study but not in this one. One participant with a very large lesion (262.8 cc) was excluded from the earlier study because large lesions are typically uninformative in LSM, but was included in this study because their connectome data may be informative.
